# Effect of antiseptic gels in the microbiologic colonization of the suture threads after oral surgery

**DOI:** 10.1038/s41598-020-65007-y

**Published:** 2020-05-20

**Authors:** Samuel Rodríguez Zorrilla, Andrés Blanco Carrión, Abel García García, Pablo Galindo Moreno, Xabier Marichalar Mendía, Rafael Seoane Prado, Antonio J. Pérez Estévez, Mario Pérez-Sayáns

**Affiliations:** 1 Oral Medicine, Oral Surgery and Implantology Unit (MedOralRes). School of Medicine and Dentistry, University of Santiago de Compostela, Santiago de Compostela, Spain; 2 Oral Medicine, Oral Surgery and Implantology Unit (MedOralRes). School of Medicine and Dentistry, University of Santiago de Compostela, Santiago de Compostela, Spain; 3 Oral Medicine, Oral Surgery and Implantology Unit (MedOralRes). School of Medicine and Dentistry. Health Research Institute of Santiago (IDIS), Santiago de Compostela, Spain; 4 Department of Oral Surgery and Implant Dentistry, University of Granada, Granada, Spain; 5 Department of Stomatology II. School of Medicine and Dentistry, Leioa, Bizkaia Spain; 6 Microbiology Unit. School of Medicine and Dentistry, University of Santiago de Compostela, Santiago de Compostela, Spain; 7 Microbiology Unit. School of Medicine and Dentistry, University of Santiago de Compostela, Santiago de Compostela, Spain; 8 Oral Medicine, Oral Surgery and Implantology Unit (MedOralRes). School of Medicine and Dentistry. Health Research Institute of Santiago (IDIS), Santiago de Compostela, Spain

**Keywords:** Maxillofacial surgery, Infection control in dentistry, Dental biofilms

## Abstract

Three different bioadhesive gels were evaluated in a double-blind randomized clinical trial in which microbial growth in the suture thread was assessed following post-surgical application of the aforementioned gels. Also assessed in this trial were, the intensity of post-surgical pain as well as the degree of healing of the patients’ surgical wounds. A total of 21 patients (with 42 wisdom teeth) participated in this trial. Chlorhexidine gel, chlorhexidine-chitosan gel, and hyaluronic acid gel were evaluated, with a neutral water-based gel serving as the control agent. The aerobic and facultative anaerobic bacterial recovery on blood agar was lower in the placebo group than in the experimental groups. The most significant difference (p = 0.04) was observed in the chlorhexidine-chitosan group. in which the growth of Blood Agar and Mitis Salivarius Agar was significantly higher than in the placebo group. The intensity of post-surgical pain was very similar among all the groups. Significantly better healing rates were observed in the patients treated with chlorhexidine-chitosan gel when compared with those who used the placebo gel (p = 0.03), and in particular when compared with those patients who used hyaluronic acid gel (p = 0.01). Through our microbiological analyses, we were able to conclude that none of the bioadhesive gels tested resulted in beneficial reductions in the bacterial/fungal populations. However, the healing rates of patients who were treated with chlorhexidine-chitosan were better than those of the patients who used either the placebo gel or the hyaluronic acid gel.

## Introduction

The lower third molars are the most commonly impacted teeth, with a calculated impaction incidence of 33% of the population^1–3^. Complications arising from impaction are very frequent, and in fact, pericoronitis accounts for approximately 50% of indicated tooth extractions. The remaining 50% of all extractions are indicated due to cavities in the second molar; for orthodontic reasons; or because of the presence of cysts or non-infection-related pain^4,5^. As such, the surgical removal of the third molars is one of the most frequently conducted oral surgery procedures.

The initial phase in the healing of a wound usually consists of wound closure through suturation. The most commonly used suture threads in third molar surgical procedures are 3–0 or 4–0 braided natural suture silk^6^. These particular threads are favoured because of their capacity to retain tensile strength^7,8^; their acceptable tissue reaction^9^; and also due to the fact that they are excellent value for the money.

The fact that the sutures used in impacted third molar extractions are susceptible to the adhesion of pathogenic bacteria^10^ is considered a risk factor^11^ for the healing of surgical wounds. Microbial adhesion and accumulation on the sutures inside the mouth may act as a focus of odontogenic infection^12^. Said infections can be caused by both aerobic and anaerobic bacteria, including species of the *Fusobacterium, Peptostreptococcus, Prevotella, Porphyromonas, Streptococcus, and Bacteroides* genera, or yeasts such as *Candida albicans*^13^.

The use of oral antiseptics before and after the surgical procedure is an efficient way of reducing the microbiota and of preventing post-surgical complications^14,15^. In addition, oral antiseptics help to prevent bacteria spreading through the blood^16^, thus decreasing the risk of bacterial endocarditis^17,18^.

It is now widely accepted that, out of all of the antimicrobial agents which are used to reduce the number of microorganisms in the mouth, chlorhexidine digluconate is the safest and most efficient option^19–21^. However, there are a number of known adverse effects of chlorhexidine digluconate. These include the formation of stains on teeth and dental prostheses^22,23^; dysgeusia and ageusia^24,25^; parotid enlargement (which depends on the exposure period)^26^; and the desquamation of the epithelium of the oral mucosa^27^. Chlorhexidine is primarily used in mouthwashes and, given that it comes into contact with all areas of the mouth, it has been associated with the aforementioned complications^28,29^.

Chlorhexidine can also be used in different kinds of gels. Recent studies have shown that the application of chlorhexidine gel following the surgical management of impacted third molars can significantly reduce infectious complications^30^.

In recent years, laboratories have taken steps to improve the substantivity of chlorhexidine; developing formulations for increased adhesion to the mouth’s surface, and these have been attained through the use of agents with bioadhesive properties^31^.

Decker *et al*.^32^ observed an important synergistic effect when combining chlorhexidine and chitosan, as this resulted in bacterial reduction. Chitosan is a polysaccharide derived from chitin which can be found in the arthropod cuticle; in marine fauna such as the squid; and in the cell wall of fungi^34^. This biomolecule displays interesting bioadhesive properties on oral tissue^33^.

Chitosan boasts antibacterial^35–37^ and antifungal^38^ properties, and is known for its high degree of biocompatibility^38–40^. Moreover, it has previously been described in the scientific literature as a wound-healing agent^39-43^; a drug delivery system for the slow release of different topical^44–47^ and systemic drugs^46,48^; a hemostatic agent^49,50^; and biomaterial for bone^51^ and cartilage^52,53^ regeneration. In addition, the use of chitosan membranes for guided bone regeneration has been considered^54^.

Due to its excellent bioadhesive properties and its high adherence to oral surfaces, chitosan is considered as the perfect vehicle for permitting increased substantivity, and likewise it is used to prolong the release of different oral therapeutic agents^55^ such as chlorhexidine.

It has also been demonstrated that hyaluronic acid gel offers a similar healing-inducing effect and antimicrobial activity. Hyaluronic acid is a glycosaminoglycan which is found in connective tissue, and which boasts a wide range of healing properties during the tissue creation and repair processes, including cell migration and differentiation^56^. Recent studies have indicated that it also plays an important role in the healing process of both soft and hard tissues^57,58^. In addition to its well-documented regenerative properties^59^, its bacteriostatic effect on numerous oral bacteria such as *Streptococcus mutans, Porphyromonas gingivalis, Prevotella oris, Actinobacillus actinomycentencomitans, Staphylococcus aureus* and other bacteria that are usually found in the oral flora^60,61^ has also been demonstrated. This effect depends on the molecular weight of hyaluronic acid and its concentration in the product. Hyaluronic acid is also effective against colonization by *Candida albicans*^62^. It also has analgesic properties^63^ and is known to protect the treated area from contaminants. It does so by creating a barrier to protect the wound from macromolecules and other substances that could be used by bacteria as a substrate^64^.

The aim of this study was to evaluate the use of different bioadhesive gels by assessing the growth of bacteria and fungi present on the suturing thread; the intensity of post-surgical pain; and the degree of healing of surgical wounds following the application of these different post-surgical bioadhesive gels.

## Material and Methods

### Type of study

In order to attain the study objective, a double-blind randomized clinical trial was performed. The study protocol was approved by the Clinical Research Ethics Committee of Galicia, and was assigned the reference code 2014/337. This project did not receive funding from any private entity. The authors declare that there are no conflicts of interest. The clinical trial was registered on ClinicalTrials.gov (ID: NCT03188289) on the 15 June 2017.

### Study population

A total of 21 patients from the Faculty of Medicine and Odontology of the University of Santiago de Compostela participated in the study from 15 January 2014 to 15 July 2014. The participating patients visited the Clinic of Oral Medicine, Oral Surgery, and Implantology for the surgical extraction of, at minimum, both of their inferior wisdom teeth.

### Inclusion and exclusion criteria

After the clinical history of each patient who attended the clinic for the surgical removal of the lower third molars was obtained, participants underwent an oral examination before their compliance with the following inclusion criteria was thoroughly evaluated; age (between 18 and 39 years); good general health; availability during the study period; no current or pending treatments during the study period; the patient’s acceptance and compliance with the prescribed oral hygiene instructions; and his or her commitment not to use any antiseptic mouthwash or toothpaste during the study period. The exclusion criteria were as follows: use of antimicrobial mouthwash or toothpaste during the period in which the sutures remain in the patient’s mouth; a diagnosis of diabetes; a diagnosis of a degenerative disease; the patient is a smoker; deficient oral health (cavities, periodontal disease, pathologies of the oral mucosa, etc.); use of prosthetic or orthodontic devices; an amoxicillin allergy; pregnancy or lactation.

Patients who did not meet these criteria were excluded from the study, but did continue to receive the surgical treatment that had previously been planned for them in the Clinic of Oral Medicine, Oral Surgery, and Implantology. Patients who met the inclusion criteria were able to voluntarily choose to participate in the study, or to refrain from doing so.

### Randomization

All of the patients who agreed to participate in the study and who signed informed consent forms were randomly assigned to one of three groups. The principal researcher generated a randomization sequence in which the participants were selected and assigned to the different treatment groups.

In order to do this, a table containing three columns (A, B, and C) was created. The name of each patient was written on a small piece of paper which was then placed in a completely opaque bag. The contents of the bag were shuffled before the names were randomly drawn. Each name drawn was assigned to one of groups, alternating among the three groups, which were assigned the letters A, B, and C. In this manner three groups, each containing seven participants, were formed. The names of the three gels which were to be used in this study were then placed in the same bag. The name of the first gel that was drawn was assigned to group A; the second to group B; and the third to group C.

### Study protocol

#### Surgical planning

All of the patients who agreed to participate in the study and who signed the informed consent forms and the specific form for oral surgery were then each given an individual appointment.

The appointments for study participants were scheduled for different dates and times in the Clinic of Oral Medicine, Oral Surgery, and Implantology of the Faculty of Odontology of the University of Santiago de Compostela exactly as would have been done if they had not agreed to participate in the study.

The choice of which of the two inferior wisdom teeth to remove first (i.e., the right or the left) was made by flipping a coin. Prior to each surgical intervention, the clinical and radiographic parameters were examined in order to assess the perceived difficulty of the tooth extraction. The analyzed parameters were the degree of eruption; the facial landmarks^65,66^(i.e., the distance from the tragus to the corner of the mouth, between the mandible and the external corner of the eye, between the corner of the lip and the mandible, the inter-incisal distance; and both the Pell and Gregory^67^, and Winter^68^ radiographic classifications.

Upon completion of the surgery, the wound was sutured using 4–0 braided natural silk and the patient was given instructions regarding their post-surgical care.

#### Post-surgical care and gels under study

The participants were prescribed 750 mg of Amoxicillin every eight hours for seven days, and 600 mg of ibuprofen every eight hours for three days. If the patients continued to experience pain after the initial three-day prescription, the same dose of ibuprofen could be administered as needed. Each patient was also provided with a coded sterile container containing 20 ml of one of the bioadhesive gels under study:

- Chlorhexidine 0.2% gel (Bexident Gel Gingival ®)
- Chlorhexidine 0.2% gel + Chitosan (Bexident Post ®)
- Hyaluronic acid gel (ODDENT ®)

The patients were informed that this was the only antiseptic and healing-inducing gel that they were permitted to use during the seven-day period before the sutures in their mouths were removed.

The antiseptic gels were allocated randomly in the “double-blind” manner described above. Thus, neither the person performing the microbiological analysis nor the patient was aware of which treatment gel was being used in each instance.

The patients were instructed to apply the gel to the wound area and to the suture thread using a cotton swab, three times a day after brushing their teeth.

#### First control visit and suture removal

Seven days after the surgical procedure had been performed on each of the patients, he or she visited the Clinic in order for the suture threads to be removed and for the general condition of the surgical wound to be assessed. Facial swelling and trismus measurements as well as intraoral pictures were taken in order to determine the extent of healing. In addition, patients were asked to use a visual analog pain scale to evaluate their overall pain level during the seven-day period following the surgical procedure. Approximately one cm of the surgical thread that was in contact with the patient’s mouth was collected and then preserved in a PBS (phosphate-buffered saline) medium (Gibco, Waltham, USA) for bacterial preservation. This sample was subsequently sent to the Faculty of Medicine’s microbiology laboratory, where the microbiological analysis described below was performed.

#### Creation of the control group

In order to create the control group, the third contralateral molar was extracted from these patients’ mouths in the exact same manner as in the study group. The only difference was in the bioadhesive gel that was used after the surgical procedure was performed.

Thus, for the control group, a neutral water-based gel without any active ingredients was used. This will subsequently be referred to as the “placebo gel”. Its brand name is HISPAGEL 200 ® (Acofarma, Madrid, Spain).

#### Organizational chart and evaluation of clinical parameters

In each experiment, the patient visited the clinic on two separate occasions (day zero: day of surgery, and day seven: suture removal). This protocol was followed for both experiments.

In total, each patient visited the clinic on five different occasions. During their first visit, their clinical history was taken, the procedure was explained, and they were offered the possibility of participating in the study. If the patient gave their consent for their participation in the study, four more visits were scheduled in order for each lower wisdom tooth to be extracted and for the surgical sutures to subsequently be removed.

The same examiner collected all the samples, took the intraoral pictures of the wound after the sutures had been removed, and gathered the data recorded from the visual analog pain scale as indicated by the patient.

The clinical parameters were also analyzed, and their association to the microbiological parameters was verified.

For the subjective evaluation of post-surgical pain, a visual analog pain scale ranging from 0 to 10 was used, where 0 represented “no pain” and 10 “the worst pain imaginable”.

In order to assess the quality of healing, and given the lack of existence of a validated scale in the scientific literature on the healing of oral mucosa, it was decided to follow the work of Bloemen et. al.^69^ on wound healing, in which it is demonstrated that there are no significant differences between subjective assessment and assessments by means of computer software. Therefore four levels of healing were established: 1. Erythema present in operated area with signs of inflammation and infection. 2. Erythema present in operated area without any signs of infection. 3. Normal colouring in operated area and initial epithelialization. 4. Normal colouring in operated area and advanced epithelialization.

### Microbiological analysis

**Figure 1:**
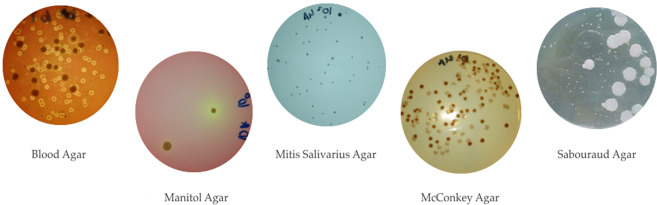
Photograph of the bacterial growth in the different culture media. (Attached file).

The samples were analyzed in the Microbiology Laboratory of the Parasitology Department of the Faculty of Medicine of the University of Santiago de Compostela. The collected samples of suture thread were immersed in phosphate-buffered saline solution (PBS) immediately after their removal and these were kept refrigerated until their microbiological analysis. The samples were shaken vigorously for two minutes in order to separate the bacteria from the suture thread, and in order to obtain a homogeneous suspension. 10-fold serial dilutions in saline were subsequently prepared (10^–1^ to 10^–5)^. Aliquots (50 microliters) of each dilution were plated in Petri dishes containing the different culture media (Fig. 1): Assays were performed in duplicate. The culture media used were: 1.) Columbia Blood agar (Liofilchem®, Italy) for the growth of aerobic and facultative anaerobic microorganisms; 2.) Mitis salivarius agar with 0.2 IU/ml of bacitracin and 15% sucrose (MSB) (Liofilchem®, Italy) for the growth of *Streptococcus mutans*: 3.) Mannitol agar (Liofilchem®, Italy) for the growth of *Staphylococcus spp*.; 4.) MacConkey agar (Liofilchem®, Italy) for the growth of *Enterobacteriaceae* and *Pseudomonas spp*.; 5.) Sabouraud dextrose agar (Liofilchem®, Italy) containing 0.1 mg/ml of chloramphenicol for the growth of *Candida spp*.

The blood agar, mannitol agar, MacConkey agar, and Sabouraud agar plates were aerobically incubated at 37 °C for 24–48 hours. The MSB agar plates were incubated in 5% CO_2_ at 37 °C for 48–72 hours.

After incubation, the colony forming units (CFUs) were counted and the total number of CFUs per milliliter of initial solution was calculated.

### Safety assessment

Patient discomfort and the other risks inherent to this study were considered to be equivalent to those of normal clinical practice. Therefore, taking out a specific insurance policy for this purpose was not considered necessary.

### Statistical analysis

In order to calculate the sample size, the average number of patients visiting the clinic annually for surgical extractions which included the third molar was used. With an average of 1000 patients per year, with 15% of these patients presenting with third molar pathology, and with a standard deviation of five, the inclusion of 42 third molars in this study results in a statistical power of 98%. These calculations were performed using Epidat 4.2 (SERGAS Galician Healthcare Service, Galicia, Spain). The data were analyzed using the SPSS (IBM SPSS Statistics 22 for MAC OX) predictive analysis software. The microbiological results and the clinical analyses were initially subjected to the Kolmogorov-Smirnov goodness-of-fit and the Shapiro-Wilk normality distribution test. If the data distribution resulted normal, the Student’s t-test was then used to perform a pairwise comparison. In the case of non-normal distribution, the Mann-Whitney U test was applied. The results were considered to be statistically significant when p ≤ 0.05.

### Ethical approval

All procedures performed in studies involving human participants were in accordance with the ethical standards of the **Clinical Research Ethics Committee of Galicia recorded under code 2014/337**, and with the 1964 Helsinki declaration and its subsequent amendments or comparable ethical standards.

### Informed consent

Informed consent was obtained from all individual participants involved in the study.

## Results

### Position of the wisdom teeth

A total of 21 patients participated in this study: 5 men (23.8%) and 16 women (76.2%). The average age of the patients in this study was 24.5 years, with a standard deviation of 4.1 years and an age range from 18 to 37 years. With regards to the degree of eruption, 52.4% of the wisdom teeth were unerupted, followed by 33.33% that were partially erupted, and only 14.3% of the extracted wisdom teeth had completely erupted at the time of surgery. The spatial orientation of the wisdom teeth was assessed using Winter’s classification. According to this classification, the vertical position was predominant, accounting for 50% of the 42 analyzed wisdom teeth, while 45.2% of wisdom teeth were in a mesioangular position, and less than 5% were in a distoangular position. In this study, no wisdom teeth were found in horizontal, transversal or inverted positions. According to the Pell and Gregory classification, all of the positions, (except for positions 2 C and 3 C) appeared in different proportions. Position 2 A was predominant, and this was present in more than 1 in 4 patients, (26.2%), and the incidence of positions, 1 A and 2B each appeared in 19.1% of patients respectively. Positions 2 C and 1 C accounted for 11.9% and 7.1%, respectively, while position 3 A appeared in only one of the studied cases, representing just 2.3% of the total sample. The frequency of these variables is shown in Table 1.

**Table 1:** Degree of eruption and classification of the position of the wisdom teeth.

		Gender		Degree of eruption			Position of the teeth according to Winter’s classification			Position of the teeth according to the Pell and Gregory classification							Total
		Female	Male	Not erupted	Partially erupted	Erupted	Mesioangular	Distoangular	Vertical	1 A	2 A	3 A	1B	2B	1 C	2 C	
**Bioadhesive gel**	**Placebo**	16 (76.2%)	5 (23.8%)	11 (52.4%)	7 (33.3%)	3 (14.3%)	9 (42.9%)	2 (9.5%)	10 (47.6%)	3 (14.3%)	6 (28.6%)	1 (4.8%)	3 (14.3%)	4 (19.0%)	2 (9.5%)	2 (9.5%)	21 (100%)
	**Chlorhexidine**	5 (71.4%)	2 (28.6%)	4 (57.1%)	2 (28.6%)	1 (14.3%)	4 (57.1%)	0 (0.0%)	3 (42.9%)	1 (14.3%)	2 (28.6%)	0 (0.0%)	1 (14.3%)	1 (14.3%)	0 (0.0%)	2 (28.6%)	7 (100%)
	**Chlorhexidine-chitosan**	5 (71.4%)	2 (28.6%)	3 (42.9%)	4 (57.1%)	0 (0.0%)	3 (42.9%)	0 (0.0%)	4 (57.1%)	3 (42.9%)	1 (14.3%)	0 (0.0%)	1 (14.3%)	1 (14.3%)	1 (14.3%)	0 (0.0%)	7 (100%)
	**Hyaluronic acid**	6 (85.7%)	1 (14.3%)	4 (57.1%)	1 (14.3%)	2 (28.6%)	3 (42.9%)	0 (0.0%)	4 (57.1%)	1 (14.3%)	2 (28.6%)	0 (0.0%)	1 (14.3%)	2 (28.6%)	0 (0.0%)	1 (14.3%)	7 (100%)
**Total**		32	10	22	14	6	19	21	21	8	11	1	6	8	3	5	42

It was determined that the classification of the degree of inclusion by means of the Pell and Gregory scale or Winter’s scale was consistent with the study’s clinical and microbiological results.

### Analysis of microbiological growth

No significant differences were detected in the mannitol, Sabouraud, and MacConkey agar media CFU counts between the placebo and any of the three experimental gels. Nevertheless the *Streptococcus mutans* counts from gels containing chlorhexidine (alone, or in combination with chitosan) were higher than those recorded with the placebo gel. The samples from the participants who used the chlorhexidine-chitosan gels showed not only higher *S. mutans* counts (p = 0.004) but also significant differences in the blood agar counts compared with the placebo levels (p = 0.047). Conversely, there was no difference in the blood agar counts when using the gels containing only chlorhexidine, compared to the placebo.

In terms of bacterial recoveries from hyaluronic gel samples, there was no significant difference with either the placebo or with gels containing chlorhexidine, regardless of the medium used for recovery.

Significant differences in the bacterial recoveries in the cases of partially erupted and completely impacted wisdom teeth were observed. The CFU counts were significantly higher in partially erupted teeth in both blood agar (aerobic and anaerobic CFUs) (p = 0.030) and MSB (*Streptococcus mutans* CFUs) (p = 0.001). The CFU counts in both blood agar (p = 0.043) and MSB (0.014) cultures were significantly higher in mesioangular wisdom teeth than in distoangular wisdom teeth. Conversely, *Streptococcus mutans* CFU counts were significantly higher in mesioangular wisdom teeth than in vertical wisdom teeth (p = 0.014).

Table 2 shows bacterial growth in the different culture media depending on the gels used in the postoperative period.

**Table 2:** Bacterial growth in the different culture media depending on the gels used during the postoperative period.

		N	Mean	Standard deviation
Mannitol Agar (*Staphylococcus spp*. CFU)	Placebo	21	2.8571E + 02	7.83764E + 02
	Chlorhexidine	7	7.1429E + 02	1.25357E + 03
	Chlorhexidine- chitosan	7	2.8571E + 02	4.87950E + 02
	Hyaluronic acid	7	8.3329E + 02	1.21335E + 03
Sabouraud Agar (*Candida spp*. CFU)	Placebo	21	4.7619E + 01	2.18218E + 02
	Chlorhexidine	7	7.1429E + 01	1.88982E + 02
	Chlorhexidine -chitosan	7	2.8571E + 02	7.55929E + 02
	Hyaluronic acid	7	1.6657E + 02	3.72678E + 02
MacConkey Agar (*Enterobacteriaceae* and *Pseudomonas spp*. CFU)	Placebo	21	1.3310E + 04	4.31678E + 04
	Chlorhexidine	7	1.4500E + 04	3.02930E + 04
	Chlorhexidine-chitosan	7	4.5000E + 03	8.53913E + 03
	Hyaluronic acid	7	8.3329E + 02	1.21335E + 03
Blood Agar (Aerobic and facultative anaerobic microorganisms CFU)	Placebo	21	1.1854E + 08	2.28996E + 08
	Chlorhexidine	7	7.3643E + 07	5.90367E + 07
	Chlorhexidine-chitosan	7	3.5993E + 08	5.06851E + 08
	Hyaluronic acid	7	3.5067E + 08	6.28146E + 08
Mitis Salivarius Agar (*Streptococcus mutans* CFU)	Placebo	21	2.5345E + 05	4.93538E + 05
	Chlorhexidine	7	3.9216E + 06	6.23368E + 06
	Chlorhexidine-chitosan	7	3.6104E + 06	7.56264E + 06
	Hyaluronic acid	7	2.9121E + 05	3.49078E + 05
	Total	42	1.4306E + 06	4.12252E + 06

### Analysis of post-surgical pain

Post-surgical pain was closely correlated with the quantity of analgesics consumed by the patients. All the patients included in the study received the mandatory prescription for 3 days, and none needed any extra dosage, hence the pain level score measures by VAS was not affected by the medication. Statistical analysis indicated that, regardless of the gel applied, the level of post-surgical pain described by the patients (using the visual analog pain scale) was very similar, and no significant differences were recorded. The mean pain level was 4.18 out of 10. The lowest pain level, 3.29 out of 10, was reported following the application of chlorhexidine gel; however, this difference was not considered statistically significant.

In total, 82.6% of the patients with low pain levels showed a “good” or “very good” degree of healing. There was no significant statistical relationship between the degree of pain and the type of gel used.

The distribution of the patients’ pain thresholds, both in the study and the control group, as well as their statistical significance, are shown in Tables 3 and 4.

**Table 3:** The participants’ evaluation of pain experienced according to the Distribution of Pain Levels in the VAS assigned by the patients.

		Visual analog pain scale									
		0	1	2	3	5	6	7	8	9	10
Bioadhesive gel	Placebo	1 (4.8%)	4 (19.0%)	4 (19.0%)	2 (9.5%)	2 (9.5%)	2 (9.5%)	2 (9.5%)	1 (4.8%)	2 (9.5%)	1 (4.8%)
	Chlorhexidine	1 (14.3%)	2 (28.6%)	1 (14.3%)	0 (0.0%)	0 (0.0%)	2 (28.6%)	1 (14.3%)	0 (0.0%)	0 (0.0%)	0 (0.0%)
	Chlorhexidine-chitosan	0 (0.0%)	2 (28.6%)	0 (0.0%)	1 (14.3%)	1 (14.3%)	1 (14.3%)	0 (0.0%)	1 (14.3%)	0 (0.0%)	1 (14.3%)
	Hyaluronic acid	0 (0.0%)	2 (28.6%)	0 (0.0%)	1 (14.3%)	1 (14.3%)	2 (28.6%)	0 (0.0%)	1 (14.3%)	0 (0.0%)	0 (0.0%)
Total		2	10	5	4	4	7	3	3	2	2

**Table 4:** Descriptive data for healing and pain in each group. The level of significance for healing following the use of the different gels (Mann-Whitney test), and the level of significance for postoperative pain following the use of the different gels (t-student test) are shown in the table. SD = standard deviation.

Bioadhesive gel	Healing, mean (SD)	Visual analog pain scale, mean (SD)	Bioadhesive gel used	Level of significance for healing	Level of significance for pain
**Placebo**	2.57 (0.93)	4.29 (3.15)	**Placebo – Chx Gel**	p = 0.26	p = 0.46
**Chx**	3.00 (0.58)	3.29 (2.93)	**Placebo – Chx-Chitosan Gel**	**p** = **0.03**	p = 0.68
**Chx-Chitosan**	3.43 (0.53)	4.86 (3.44)	**Placebo – Hyaluronic acid Gel**	p = 0.45	p = 1.00
**Hyaluronic Acid Gel**	2.29 (0.76)	4.29 (2.69)	**Chx Gel – Chx-Chitosan Gel**	p = 0.17	p = 0.37
**Total**	2.74 (0.86)	4.21 (3.01)	**Chx Gel – Hyaluronic Acid Gel**	p = 0.07	p = 0.52
			**Chx-Chitosan Gel – Hyaluronic acid Gel**	**p** = **0.01**	p = 0.57

### Analysis of the degree of healing

The results from the Mann-Whitney U test for the two independent samples have been included in Table 4. The degree of healing of participants in the chlorhexidine-chitosan gel group was significantly greater than that of the participants in the placebo gel group (U = 34.500; p = 0.03). Similarly, the patients belonging to the chlorhexidine-chitosan gel group showed healing levels that were superior to those of the patients in the hyaluronic acid gel group, with an even greater difference than when compared to the placebo gel group (U = 6.000; p = 0.01).

No statistical differences were observed in the other comparisons with regard to the degree of healing.

The distribution of the degree of healing according to the bioadhesive gel used is indicated in Table 5.

**Table 5:** Distribution of healing levels according to the gel used as evaluated by the clinician in 4 levels: 1- Erythema present in operated area with inflammation and infection. 2- Erythema present in operated area without any signs of infection. 3- Normal colouring in operated area and initial epithelialization. 4. Normal colouring in operated area and advanced epithelialization.

		Degree of healing			
		Poor	Average	Good	Very Good
Bioadhesive Gel	Placebo	3 (14.3%)	6 (28.6%)	9 (42.9%)	3 (14.3%)
	Chlorhexidine	0 (0.0%)	1 (14.3%)	5 (71.4%)	1 (14.3%)
	Chlorhexidine-chitosan	0 (0.0%)	0 (0.0%)	4 (57.1%)	3 (42.9%)
	Hyaluronic acid	1 (14.3%)	3 (42.9%)	3 (42.9%)	0 (0.0%)
Total		4	10	21	7

### Analysis of facial swelling and trismus

Statistical analysis indicated that, regardless of the gel applied, the post-surgical swelling and the trismus measurements between the anatomical landmarks before the surgical procedure was performed, and then seven days afterward, were not statistically different.

The average values before and after surgery are reflected in Table 6.

**Table 6:** Swelling and average trismus measurements before and after the surgical procedure.

	Day 0 (Prior to Surgery)	Day 7 (Suture removal)
Distance from the tragus to the corner of the mouth (cm)	8.79 ± 0.71	9.03 ± 0.37
Distance between the mandible and the external corner of the eye (cm)	8.25 ± 0.62	8.45 ± 0.43
Distance between the corner of the lip and the mandible (cm)	11.03 ± 0.8	11.27 ± 0.17
Inter-incisal distance (cm)	4.40 ± 0.3	3.79 ± 0.7

## Discussion

In this clinical study, the antimicrobial effect of three bioadhesive gels used following the extraction of the lower third molars were assessed. Data on post-surgical pain, swelling, and the quality of healing were also evaluated.

This study confirmed the findings of Falci *et al*.^70^ regarding the prevalence of the positions of the wisdom teeth, indicating that the most common positions were vertical in Winter’s scale (50%), and position 2 in the Pell and Gregory classification (26%).

The microbiological analysis of the samples indicated that there were some significant differences in the microbiological growth on the suture thread among the different gels. The most noteworthy difference was observed in the levels of growth of CFUs (blood agar) between the chlorhexidine-chitosan and the placebo gel, with bacteria growth being significantly higher in the chlorhexidine-chitosan group. The authors believe that this could be related to the possibility that some bacteria use chitosan in their metabolism through chitosanases^71^.

The *Streptococcus mutans* recovery (Mitis salivarius agar) was lower in the placebo group than in the chlorhexidine gel and chlorhexidine-chitosan gel groups. This effect was paradoxical, given that there are studies which demonstrate that chlorhexidine, both in mouthwashes and in gels, is the substance that has the greatest effect on the *Streptococcus mutans* on dental plaque. A study by Emilson *et al*.^72^ indicated that chlorhexidine is considered the gold standard for combatting *Streptococcus mutans*. Other authors also confirmed the capacity of chitosan, in varying forms, for reducing the load of this bacterium^73,74^. However, all such studies consider the effect of the mouthwash outside of the surgical field (i.e., in a manner which is completely unrelated to the colonization of a surgical wound and its suture).

It should be noted that the final non-selective aerobic and facultative anaerobic bacteria counts in the placebo gel were significantly lower than in the chlorhexidine-chitosan gel. This was a surprising finding, given that it has previously been reported that the physical and chemical properties of this combination extend the effect of chlorhexidine in the mouth, when compared with the other gels^75^.

It was observed that, even though the placebo gel achieved a lower blood agar growth of bacteria when compared to the chlorhexidine-chitosan gel, this does not imply a direct relationship with wound healing given that, as other authors have also reported^76^, the chlorhexidine-chitosan gel managed to attain a significantly better degree of healing than the placebo gel. The effect of chitosan in favouring the healing process depends on individual host factors such as immunity, vascular parameters, and the molecules involved in bone regeneration such as RANK-RANKL-OPG^77^.

In future studies, it would be interesting to establish if any of the limited components present in the placebo gel formula could present an antimicrobial effect against the studied bacteria, given that it has been determined that it boasted certain advantages when compared with other gels (i.e., its recognized antimicrobial effect). The authors believe that this could be due to its strong adhesion to the surfaces on which it is applied, which as a consequence, prevents bacterial adhesion and the growth of biofilms.

In terms of the reduction in pain following the application of the different gels, the best rate was obtained from participants in the chlorhexidine gel group, with results of almost one point lower than the mean. However, this was not considered to be a statistically significant result. We can conclude that the mean post-surgical pain for this procedure was moderate; slightly over 4 out of 10 points. We did not observe any differences in the degree of facial swelling depending on the gel used.

The clear superiority of the chlorhexidine-chitosan gel in terms of the surgical healing state^78^ was associated with its chitosan content. This difference was observed both in the clinic and during statistical testing. The overall healing was significantly better in participants in the chlorhexidine-chitosan gel group than in those who used either the placebo gel or the hyaluronic acid gel, with p values of 0.027 and 0.011, respectively. No significant differences were observed when comparing it with the chlorhexidine gel alone, perhaps due to the sample size. Nonetheless, the mean surgical healing for chlorhexidine-chitosan was 0.83 points better than chlorhexidine alone.

No clear relationship between the microbiological parameters analyzed and the clinical aspects of pain and healing level was established.

The results which were obtained with the studied gels, in particular in terms of the healing quality, are considered to be useful when making recommendations to patients who have undergone a surgical procedure on their wisdom teeth and, by extension, to patients who have undergone general oral surgery.

## Conclusions

The microbiological analyses revealed that none of the bioadhesive gels tested led to beneficial reductions in the bacterial/fungal populations. Likewise, none of the gels resulted in reduced post-surgical pain or facial swelling. The patients who were treated with chlorhexidine-chitosan gel healed better than those who were prescribed the placebo and the hyaluronic acid gels, and therefore this must be taken into consideration when prescribing medication following a surgical procedure to remove a lower third molar tooth.
